# Supplementary material for: Therapeutic miR-21 Silencing Reduces Cardiac Fibrosis and Modulates Inflammatory Response in Chronic Chagas Disease
Source: Int J Mol Sci. 2021 Mar 24;22(7):3307. doi: 10.3390/ijms22073307 (PMC8036348; doi:10.3390/ijms22073307)
Supplement: Supplementary file 1 [file ijms-22-03307-s001.pdf]

Therapeutic miR-21 silencing reduces cardiac fibrosis and modulates inflammatory response in chronic Chagas disease

Carolina Kymie Vasques Nonaka[1-3], Gabriela Louise Sampaio[2,4], Luciana de Aragão França[1,3], Bruno Raphael Ribeiro Cavalcante[2], Katia Nunes Silva[1-3], Ricardo Khouri[2], Felipe Guimarães Torres[2], Cássio Santana Meira[2,4], Emanuelle de Souza Santos[2,4], Carolina The Macedo[2,4,5], Bruno Diaz Paredes[1-3], Vinicius Pinto Costa Rocha[2,4], Silvia Regina Rogatto[5], Ricardo Ribeiro dos Santos[2,4], Bruno Solano de Freitas Souza[1-3], Milena Botelho Pereira Soares[2,4]*

Supplementary material

Table S1. Predicted targets of miR-21-5p sorted by cumulative weighted context++ score by TargetScanHuman

Ortholog of target gene	Gene name	Total context++ score
C1orf143	chromosome 1 open reading frame 143	-0.85
ZNF367	zinc finger protein 367	-0.72
KHDC1L	KH homology domain containing 1-like	-0.71
ABHD12B	abhydrolase domain containing 12B	-0.69
CDR1as	circular RNA CDR1as	-0.68
KHDC1	KH homology domain containing 1	-0.68
HTN1	histatin 1	-0.66
KRIT1	KRIT1. ankyrin repeat containing	-0.69
IL12A	interleukin 12A (natural killer cell stimulatory factor 1. cytotoxic lymphocyte maturation factor 1. p35)	-0.65
FASLG	Fas ligand (TNF superfamily. member 6)	-0.64
FGF18	fibroblast growth factor 18	-0.64
CCL1	chemokine (C-C motif) ligand 1	-0.64
CALCB	calcitonin-related polypeptide beta	-0.57
GPR64	G protein-coupled receptor 64	-0.55
AIM1L	absent in melanoma 1-like	-0.55
MTPN	myotrophin	-0.54
PLEKHA1	pleckstrin homology domain containing. family A (phosphoinositide binding specific) member 1	-0.54
RIOK1	RIO kinase 1	-0.52
RSAD2	radical S-adenosyl methionine domain containing 2	-0.52
TRAPPC2	trafficking protein particle complex 2	-0.53
ATXN10	ataxin 10	-0.57
LUM	lumican	-0.51
RPL36A	ribosomal protein L36a	-0.51
SCML2	sex comb on midleg-like 2 (Drosophila)	-0.51
ALDH1A1	aldehyde dehydrogenase 1 family. member A1	-0.51
YOD1	YOD1 deubiquitinase	-0.75
PELI1	pellino E3 ubiquitin protein ligase 1	-0.5
ANGPTL5	angiopoietin-like 5	-0.49
TGFBI	transforming growth factor. beta-induced. 68kDa	-0.52
ARMCX1	armadillo repeat containing. X-linked 1	-0.49

B3GAT2	beta-1.3-glucuronyltransferase 2 (glucuronosyltransferase S)	-0.70
MATN2	matrilin 2	-0.48
HSD17B4	hydroxysteroid (17-beta) dehydrogenase 4	-0.49
SKP2	S-phase kinase-associated protein 2. E3 ubiquitin protein ligase	-0.47
CDK2AP1	cyclin-dependent kinase 2 associated protein 1	-0.47
NTF3	neurotrophin 3	-0.47
MRPL9	mitochondrial ribosomal protein L9	-0.47
SPDYA	speedy/RINGO cell cycle regulator family member A	-0.46
TIMP3	TIMP metallopeptidase inhibitor 3	-0.68
BEST3	bestrophin 3	-0.49
SMAD7	SMAD family member 7	-0.46
MCMDC2	minichromosome maintenance domain containing 2	-0.46
MSH2	mutS homolog 2	-0.46
SC5D	sterol-C5-desaturase	-0.46
EIF1AX	eukaryotic translation initiation factor 1A. X-linked	-0.46
SEPT10	septin 10	-0.45
RNFT1	ring finger protein. transmembrane 1	-0.45
SATB1	SATB homeobox 1	-0.55
PHF14	PHD finger protein 14	-0.58
FAM13A	family with sequence similarity 13. member A	-0.44
VSNL1	visinin-like 1	-0.44
RP2	retinitis pigmentosa 2 (X-linked recessive)	-0.44
RTN4	reticulon 4	-0.43
LZTFL1	leucine zipper transcription factor-like 1	-0.43
C9orf38	chromosome 9 open reading frame 38	-0.43
HNMT	histamine N-methyltransferase	-0.43
ARHGAP24	Rho GTPase activating protein 24	-0.43
UBE2D3	ubiquitin-conjugating enzyme E2D 3	-0.51
PPP1R3B	protein phosphatase 1. regulatory subunit 3B	-0.42
LRRC57	leucine rich repeat containing 57	-0.42
DUSP8	dual specificity phosphatase 8	-0.49
PDCD4	programmed cell death 4 (neoplastic transformation inhibitor)	-0.5
PPP1R3D	protein phosphatase 1. regulatory subunit 3D	-0.42
TPRG1L	tumor protein p63 regulated 1-like	-0.45
SOX5	SRY (sex determining region Y)-box 5	-0.43
KBTBD7	kelch repeat and BTB (POZ) domain containing 7	-0.42
RMND5A	required for meiotic nuclear division 5 homolog A (S. cerevisiae)	-0.41
BRCC3	BRCA1/BRCA2-containing complex. subunit 3	-0.41

RAB22A	RAB22A. member RAS oncogene family	-0.54
PDZD8	PDZ domain containing 8	-0.48
PDLIM5	PDZ and LIM domain 5	-0.41
OLR1	oxidized low density lipoprotein (lectin-like) receptor 1	-0.54
SLC2A4RG	SLC2A4 regulator	-0.48
SPRY1	sprouty homolog 1. antagonist of FGF signaling (Drosophila)	-0.41
RASGRP1	RAS guanyl releasing protein 1 (calcium and DAG-regulated)	-0.40
PFKM	phosphofructokinase. muscle	-0.40
S100A10	S100 calcium binding protein A10	-0.40
RALGPS2	Ral GEF with PH domain and SH3 binding motif 2	-0.51
TSPO	translocator protein (18kDa)	-0.40
GLIS2	GLIS family zinc finger 2	-0.39
KLF5	Kruppel-like factor 5 (intestinal)	-0.39
TEX12	testis expressed 12	-0.39
FAM177B	family with sequence similarity 177. member B	-0.39
BRWD1	bromodomain and WD repeat domain containing 1	-0.57
SPATA5L1	spermatogenesis associated 5-like 1	-0.39
CCDC121	coiled-coil domain containing 121	-0.39
TMEM68	transmembrane protein 68	-0.42
PDAP1	PDGFA associated protein 1	-0.40
SPRY2	sprouty homolog 2 (Drosophila)	-0.39
ELF2	E74-like factor 2 (ets domain transcription factor)	-0.38
RECK	reversion-inducing-cysteine-rich protein with kazal motifs	-0.38
PCBP1	poly(rC) binding protein 1	-0.38
C7	complement component 7	-0.39
CLDN8	claudin 8	-0.38
FNIP2	folliculin interacting protein 2	-0.38
TGM4	transglutaminase 4	-0.38
SLC16A10	solute carrier family 16 (aromatic amino acid transporter). member 10	-0.64
ST3GAL6	ST3 beta-galactoside alpha-2.3-sialyltransferase 6	-0.74
RNF32	ring finger protein 32	-0.37
ZNF728	zinc finger protein 728	-0.37

Release 7.2: March 2018

A

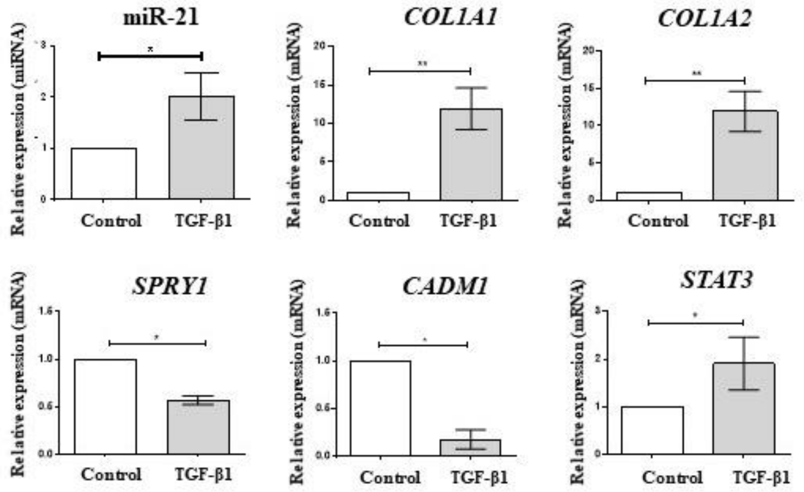

B

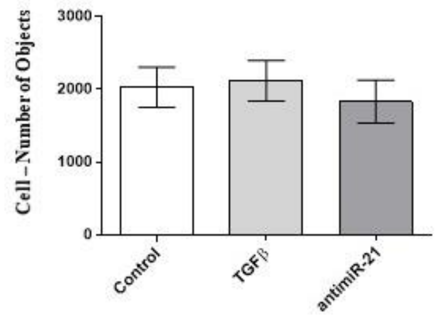

Figure S1 - MiR-21 and mRNA expression in human cardiac fibroblasts. (**A**) MiR-21, *COL1A1*, *COL1A2, SPRY1*, *CADM1,* and *STAT3* expression levels 24h after stimulation with TGF-β1, measured by RT-qPCR (Fold Change to non-stimulated control), miRNA was normalized to miR-423-3p and mRNA were normalized to *HPRT/GAPDH*. (**B**) Evaluation of cell viability by propidium iodide (PI) staining in cells treated with LNA antimiR-21 for 24 h. Results are expressed as mean ± SEM of three independent experiments. (**A**) *p<0.05, **p<0.01, Student's *t*-test.

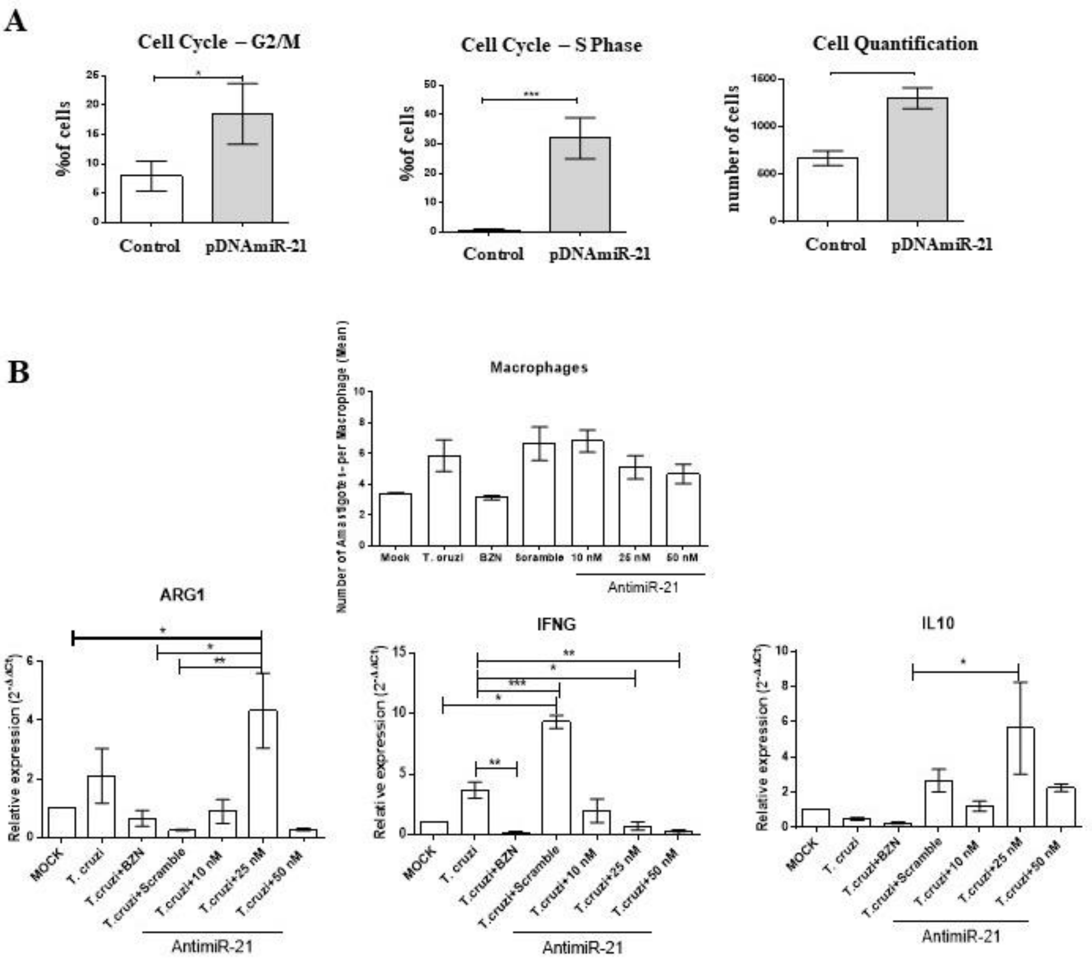

Figure S2 - MiR-21 increases proliferation of cardiac fibroblasts and *T. cruzi* infection in macrophages. **(A)** Cell cycle analysis in EDU and Hoeschst stained cells evaluated using Operetta HCT. Percentage of cells in G2/M (high DNA content) and S phases, and total cell numbers. **(B)** The mRNA expression of *Arg1*, *Ifnγ* and *Il10* in murine macrophages after *T. cruzi* infection and treated with LNA-antimiR-21. Data represent the mean ± SEM of three independent experiments, ANOVA, *$p<0.05$, **$p<0.01$, ***$p<0.001$.

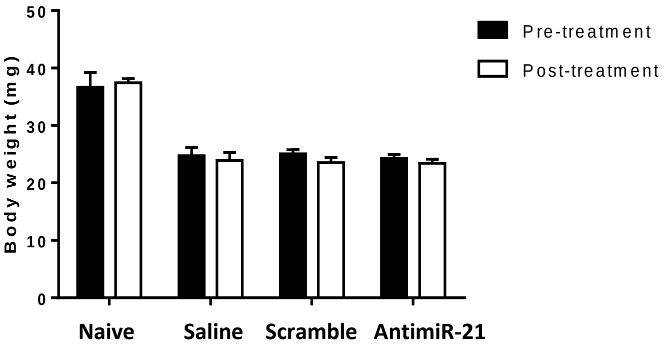

Figure S3 - Evaluation of LNA-anti-miR-21 inhibitor treatment in chronic Chagas disease model. Body weight measures before and after treatments. Data are represented as mean ± SEM, n=8 per group. ANOVA with Bonferroni comparisons.
